# Multiple tolerance defects contribute to the breach of B cell tolerance in New Zealand Black chromosome 1 congenic mice

**DOI:** 10.1371/journal.pone.0179506

**Published:** 2017-06-19

**Authors:** Nan-Hua Chang, Kieran P. Manion, Christina Loh, Evelyn Pau, Yuriy Baglaenko, Joan E. Wither

**Affiliations:** 1 Arthritis Centre of Excellence, Division of Genetics and Development, Krembil Research Institute, Toronto, Ontario, Canada; 2 Department of Immunology, University of Toronto, Toronto, Ontario, Canada; 3 Department of Medicine, University of Toronto, Toronto, Ontario, Canada; 4 Division of Rheumatology, University Health Network, Toronto, Ontario, Canada; Instituto Nacional de Ciencias Medicas y Nutricion Salvador Zubiran, MEXICO

## Abstract

Lupus is characterized by a loss of B cell tolerance leading to autoantibody production. In this study, we explored the mechanisms underlying this loss of tolerance using B6 congenic mice with an interval from New Zealand Black chromosome 1 (denoted c1(96–100)) sufficient for anti-nuclear antibody production. Transgenes for soluble hen egg white lysozyme (sHEL) and anti-HEL immunoglobulin were crossed onto this background and various tolerance mechanisms examined. We found that c1(96–100) mice produced increased levels of IgM and IgG anti-HEL antibodies compared to B6 mice and had higher proportions of germinal center B cells and long-lived plasma cells, suggesting a germinal center-dependent breach of B cell anergy. Consistent with impaired anergy induction, c1(96–100) double transgenic B cells showed enhanced survival and CD86 upregulation. Hematopoietic chimeric sHEL mice with a mixture of B6 and c1(96–100) HEL transgenic B cells recapitulated these results, suggesting the presence of a B cell autonomous defect. Surprisingly, however, there was equivalent recruitment of B6 and c1(96–100) B cells into germinal centers and differentiation to splenic plasmablasts in these mice. In contrast, there were increased proportions of c1(96–100) T follicular helper cells and long-lived plasma cells as compared to their B6 counterparts, suggesting that both B and T cell defects are required to breach germinal center tolerance in this model. This possibility was further supported by experiments showing an enhanced breach of anergy in double transgenic mice with a longer chromosome 1 interval with additional T cell defects.

## Introduction

Production of anti-nuclear antibodies (Ab) is a defining characteristic of Systemic Lupus Erythematosus (SLE). However, these patients also produce autoantibodies against a diverse array of antigens, suggesting the presence of generalized tolerance defects [1,2]. While a variety of different cellular populations could contribute to these defects, studies of lupus-prone mice indicate that altered B cell function is likely to play an important role in this tolerance disruption, and in support of this concept, several lupus risk variants that are predicted to predominantly act in B cells have been described in humans [3–5]. Because of the difficulty in studying the mechanisms that lead to perturbed B cell tolerance in humans, mouse models of lupus have been particularly informative in studying how altered expression/function of B cell expressed genes disturb B cell tolerance processes to promote autoantibody production [6–8].

Our laboratory has been studying the New Zealand Black (NZB) lupus-prone mouse model. In these mice, one of the major regions promoting anti-nuclear antibody production maps to chromosome 1 (c1), overlapping with the *Sle1* locus defined for the lupus-prone NZM2410 (NZM) mouse model [9–11]. In previous work, we produced C57BL/6 (B6) congenic mice with a series of overlapping introgressed NZB intervals of varying lengths and showed that an interval extending from 96–100 cM (170.8–181.0 Mb; c1(96–100)) was required for anti-nuclear autoantibody production. We also showed that B cells from mice with a longer NZB region encompassing the 96–100 cM interval had higher levels of activation markers, more efficient recruitment into spontaneous germinal centers, and enhanced autoantibody production as compared to B6 B cells in a mixed hematopoietic chimeric (MC) mouse with a mixture of both bone marrows [12–14]. As these changes were consistent with a breach of B cell anergy [15–18], we hypothesized that a genetic polymorphism within the c1 96–100 cM interval led to defective anergy induction.

The NZB c1 96–100 cM interval contains the *Slam* locus. It has been previously reported that the NZM *Slam* locus (derived from the NZW mouse strain) has a number of sequence polymorphisms as compared to B6 mice, and that NZB mice share some of these polymorphisms [13,19–21]. Congenic mice with the NZM *Slam* locus (*Sle1b*) have a number of disturbances of B cell tolerance, including: attenuated immature B cell signaling, receptor editing, and deletion; a breach of peripheral anergy; and altered germinal center tolerance [22–26]. While all of these processes contribute to the autoantibodies seen in these mice, it remains unclear whether NZB mice share these abnormalities and to what extent autoantibody production may be driven by the T cell functional abnormalities that are also present in these mouse strains [27–29].

In this study, we have examined these questions through the generation of c1(96–100) mice with an anti-hen egg white lysozyme (HEL) immunoglobulin (Ig) transgene alone (IgTg) or in combination with a soluble HEL (sHEL) transgene (DTg) [15,18]. We show that these mice share the same B cell central tolerance and functional abnormalities as observed for corresponding IgTg and DTg mice with the NZM Slam locus [20]. Through the generation of hematopoietic chimeric mice with a mixture of B6.CD45.1.IgTg and c1(96–100).CD45.2.IgTg bone marrow in the presence or absence of sHEL, we dissected the relative contribution of intrinsic B and T cell functional abnormalities in these mice to the breach of tolerance. Despite increased numbers of germinal center B and T cells in c1(96–100) DTg mice, B6.CD45.1 and c1(96–100).CD45.2 IgTg B cells are similarly recruited into germinal centers (GCs). However, c1(96–100) T cells demonstrated enhanced differentiation to T follicular helper (TFH) cells in DTg mice and the proportion of c1(96–100) IgTg B cells in the long-lived bone marrow plasma cell compartment was significantly increased, as compared to B6 IgTg T and B cells, respectively. Consistent with an important role for T cells in this breach of tolerance, mice with a larger c1 interval from 70–100 cM (124.6–181.0 Mb; c1(70–100)) that leads to further disruption of T cell function had augmented anti-HEL antibody production. These findings indicate that breach of tolerance in the NZB c1 strain results from an interplay between the intrinsic B and T cell defects in these mice.

## Materials and methods

### Ethics statement

Mice were housed in a Canadian Council on Animal Care approved facility at the Krembil Research Institute in the Krembil Discovery Tower, part of the University Health Network. All experiments performed in this study were approved by the Animal Care Committee of the University Health Network (Animal Use Protocol #123).

### Mice

C57BL/6 (B6) mice expressing transgenes encoding sHEL (ML5) or IgM/IgD heavy and light chains specific for HEL (MD4; IgTg) were purchased from The Jackson Laboratory (Bar Harbor, ME). Transgenes (ML5 or MD4) were backcrossed onto B6 congenic mice with NZB c1 intervals extending from 96–100 cM and 70–100 cM that were previously generated in the laboratory [14]. DTg mice that expressed both Ig and sHEL transgenes were produced by intercrossing IgTg and sHEL mice. All mice were housed in standard microisolator chambers, fed a normal rodent diet, and euthanized by CO_2_ or cervical dislocation.

### ELISA and ELISpot assays

Levels of anti-HEL IgM^a^, anti-HEL IgG, anti-ssDNA IgG, and total IgM^a^, IgM^b^, or IgG Ab were measured by ELISA, as previously described [30]. Briefly, plates were coated with HEL, ssDNA or goat anti-mouse IgM or IgG (Jackson ImmunoResearch, West Grove, PA), blocked with 5% BSA in PBS and incubated with serum for 1 hr at room temperature. Plates were then incubated with either alkaline phosphatase-conjugated anti-IgM or IgG (Southern Biotech, Birmingham, AL), or biotinylated IgM^a^ or IgM^b^ (BD Biosciences). Plates using biotinylated antibodies were further incubated with alkaline phosphatase-conjugated streptavidin (Southern Biotech). Ab levels were then revealed using *p*-nitrophenyl phosphate disodium hexahydrate substrate (Sigma-Aldrich) and measured at 405nm. All serum samples were diluted at 1:100 for anti-HEL and anti-ssDNA ELISAs, 1:3000 for total IgM^a^/IgM^b^ or 1:2000 for total IgG. Ab-producing cells were quantified by ELISpot. Briefly, 96-well Multiscreen-HA plates (Millipore, Bedford, MA) were coated with PBS or HEL (50 μg/ml; Sigma-Aldrich). Following blocking, freshly isolated splenocytes were plated at 10^6^ cells/well and incubated for 48 hours at 37°C. The cells were washed with PBS/Tween 20 and biotinylated anti-mouse IgM^a^ was added to the wells for 2 hours at room temperature. Following washing, alkaline phosphatase-conjugated streptavidin (BD Biosciences) was added, the plates were washed again, and then substrate was added (Fast5-bromo-4-chloro-3-indolyl phosphate/nitroblue tetrazolium; Sigma-Aldrich). Individual blue spots reflecting Ab-producing cells were visualized using a stereomicroscope.

### Immunofluorescence staining of splenic tissue sections

Spleens were snap-frozen in OCT compound (Sakura Finetek, Torrance, CA) at the time of sacrifice. Cryostat spleen sections (5 μm) were fixed in acetone, washed with PBS, and blocked with PBS/5% fetal bovine serum. Spleen sections were stained with: FITC-anti-IgD or -anti-CD45.1/CD45.2 (BD Biosciences); biotin-conjugated PNA (Sigma-Aldrich), HEL, anti-CD45.1/CD45.2, anti-B220 (BD Biosciences), anti-IgM^a^, or anti-CD4 (Cedarlane Laboratories); and PE-anti-IgM^a^ or -anti-CD4 (BD Biosciences). Biotinylated Ab staining was revealed with rhodamine (tetramethylrhodamine)-conjugated streptavidin (Molecular Probes) or 7-amino-4-methylcoumarin-3-acetic acid-conjugated streptavidin (AMCA, Jackson ImmunoResearch) as a secondary reagent. Stained sections were mounted with Fluoro-Gel (Electron Microscopy Sciences), and tissue fluorescence was visualized using a Zeiss Axioplan 2 imaging microscope (Zeiss, Oberkochen, Germany).

### Flow cytometry staining and analysis

Erythrocyte-depleted spleen or bone marrow cells were stained and analyzed as previously described [31]. Half a million RBC-depleted splenocytes were incubated with mouse IgG (Sigma-Aldrich) for 15 min prior to staining with various combinations of directly-conjugated mAbs. The following directly conjugated mAbs were purchased from BD Biosciences (San Diego, CA): FITC-conjugated anti-CD86 (GL1), -CD95 (Jo2), -CD4 (RM4-5), -CD3 (145-2C11), -CD44 (IM7), -CD62L (MEL-14), -CXCR5 (2G8), and -GL7 (GL7); biotin-conjugated anti-B220 (RA3-6B2), -IgM^a^ (DS-1), -CD24 (M1/69), -CD23 (B3B4), -CD138 (281–2), -CD19 (1D3) and -CD21 (7G6); allophycocyanin-conjugated anti-IFN-γ (XMG1.2); Alexa Fluor488-conjugated anti-IL17A (TC11-18H10); and PE-anti-IL21 (4A9). Biotinylated peanut agglutinin (PNA) was purchased from Sigma-Aldrich (St. Louis, MI) and biotinylated polyclonal rabbit anti-HEL Ab was purchased from Rockland (Gilbertsville, PA). Isotype controls were purchased from Caltag (Buckingham, UK). Allophycocyanin-, PE-, or PerCP-conjugated streptavidin (BD Biosciences) were used to reveal biotinylated Ab staining. Dead cells were excluded by staining with propidium iodide (PI, Sigma-Aldrich), 0.6 μg/ml. Stained cells were acquired and analyzed using a BD FACSCalibur and FACSDiva software, or using a BD LSRII flow cytometer and FlowJo software (TreeStar, San Carlos, CA).

### In-vitro B cell proliferation, CD86 up-regulation, and PI3 kinase activity assays

B cells were purified using a mouse B cell negative isolation kit (Dynal Biotech, Oslo, Norway). 5×10^4^ B cells were cultured in triplicate in media (RPMI plus 10% FBS, non-essential amino acids, L-glutamine, β-mercaptoethanol, and penicillin-streptomycin) alone or with a submitogenic concentration of LPS (50 ng/ml) and various concentrations of HEL (0–1000 ng/ml) (Sigma-Aldrich). B cell proliferation was measured by [^3^H]-thymidine incorporation. For induction of CD86 expression, 5×10^5^ sorted B cells or T cell-depleted splenocytes from IgTg or DTg mice were incubated in media alone or containing various concentrations of HEL or anti-IgM F(ab’)_2_ (10 μg/ml, Jackson ImmunoResearch) at 37°C overnight. The cells were then stained with anti-B220, -IgM^a^, and -CD86 mAb, prior to analysis by flow cytometry. To quantify phosphatidylinositol 3,4,5,-tri phosphate (PI (3,4,5) P3) production, splenocytes were stimulated with anti-IgM F(ab’)_2_ (10 μg/ml) for 5 minutes, fixed with 1% paraformaldehyde, stained with anti-B220, permeabilized with Cytofix/Cytoperm (BD Biosciences), and then stained intracellularly with biotinylated anti-PI(3,4,5) P3 Ab (Echelon Biosciences, Salt Lake City, UT).

### Measurement of Ca^2+^ mobilization and B cell apoptosis assay

Immature naïve B cells, which were generated from bone marrow cells cultured with IL-7 (10 ng/ml; R&D Systems, Minneapolis, MN) for 5 days, were incubated in serum-deprived conditions for 1 hour in Tyrode’s buffer, and then labeled with 5 μM Indo-1 AM (Molecular Probes) and 0.03% pluronic F-127 (Molecular Probes) for 30 min at 37°C. After washing, the cells were rested at 37°C for 10 min. Baseline events were acquired for 1 min before addition of (Fab’)_2_ goat anti-mouse IgM (20 μg/ml) using an LSRII flow cytometer. For measurement of B cell apoptosis, 5×10^5^ negatively sorted splenic B cells or bone marrow-derived immature naïve B cells were cultured overnight in media alone or with various concentrations of HEL or anti-IgM F(ab’)_2_ (Jackson ImmunoResearch) (10 μg/ml), or in media supplemented with avidin alone (20 μg/ml, Sigma) or together with biotinylated anti-IgM F(ab’)_2_ (10 μg/ml, Jackson ImmunoResearch). Following culture, cells were stained with anti-B220 and -IgM^a^ Abs. The proportion of apoptotic cells was determined through positive staining with Propidium Iodide (PI) and analyzed by flow cytometry. The percent surviving cells was calculated as the surviving cells in stimuli divided by the surviving cells in media alone.

### Bone marrow mixed hematopoietic chimeric mice

For generation of mixed hematopoietic chimeric mice, 1×10^7^ T-cell-depleted bone marrow cells from six-week-old female B6.CD45.1.IgTg and c1(96–100).IgTg (CD45.2 allotype) mice were mixed in a 1:1 ratio and injected intravenously into eight-week-old (B6.CD45.1 x c1(96–100))F1 sHEL or nTg recipient mice that had been irradiated with 1050 rad. After 12 weeks, the mice were sacrificed and cells from spleen and bone marrow were analyzed.

### Cytokine production by activated CD4^+^ T cells

Purified CD4^+^ T cells were isolated from splenocytes using a Dynal Mouse CD4 Negative Isolation Kit, resuspended in media and stimulated for 4 hours with PMA (50 ng/ml, Sigma-Aldrich) and ionomycin (1 μg/ml, Sigma-Aldrich) in the presence of GolgiStop (BD Biosciences) at 37°C. The cells were then stained for surface T cell (CD3, CD4) markers, fixed, permeabilized with Cytofix/Cytoperm (BD Biosciences), and stained with directly conjugated mAbs for detection of intracellular cytokines, including IL-21, IL-17 and IFN-γ.

### Statistics

For comparisons of differences between groups of mice, a Mann–Whitney U non-parametric test or a pair-study non-parametric test was used. The asterisk indicates a p value of <0.05 (*), <0.001 (**), <0.0001 (***).

## Results

### c1(96–100) DTg mice breach tolerance to HEL

**Fig 1:**
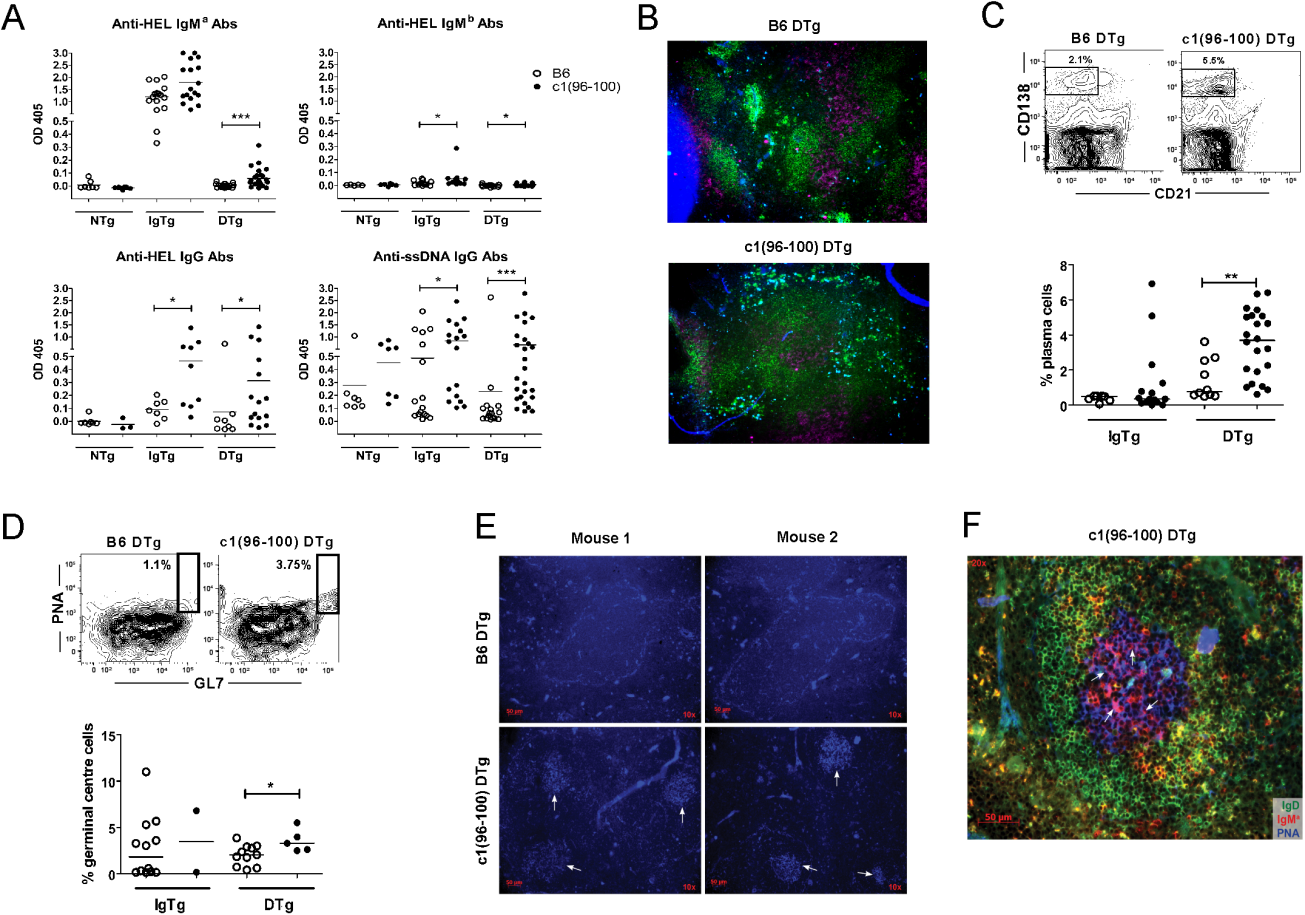
Increased levels of IgM and IgG anti-HEL Abs in c1(96–100) DTg mice indicate a breach of B cell anergy. (A) Scatter plots showing serum levels of anti-HEL and anti-ssDNA Abs from B6 (open circles) and c1(96–100) (filled circles) mice. IgM^a^, IgM^b^, or IgG anti-HEL and IgG anti-ssDNA Abs from 3- to 6-month-old mice (mean age: B6 DTg = 4.17 ± 0.77 months; c1(96–100) DTg = 4.64 +/- 0.77 months) were measured by ELISA. (B) Immunofluorescent imaging of HEL-binding plasmablasts within the B cell follicle, bridging channels, red pulp, and marginal zones of B6 and c1(96–100) DTg mice. Spleen sections (5 μm) were stained with anti-IgD (green), anti-CD4 (purple), and biotinylated-HEL, with streptavidin-AMCA as the secondary stain (blue). Magnification x10. (C) Flow plots showing the regions used to gate antibody-secreting cells (CD138^hi^CD21^-^) in the bone marrow. Scatter plots show the proportions of long-lived plasma cells in the bone marrow of c1(96–100) DTg mice (filled circles) compared to B6 DTg mice (open circles). (D) Flow plots showing the regions used to gate germinal center (PNA^+^GL7^+^) B cells in the spleen. Scatter plots show the proportions of germinal center B cells in c1(96–100) DTg mice (filled circles) compared to B6 DTg mice (open circles). (E) Immunofluorescent imaging of GC (indicated by arrows) in the spleens of two representative DTg mice per strain. Spleen sections have been stained with biotinylated-PNA followed by streptavidin-AMCA (blue). Magnification x10. (F) A representative GC at higher magnification (x20) from a c1(96–100) DTg mouse stained as in (E) together with FITC anti-IgD (green) and PE-anti-IgM^a^ (red). For scatterplots, each symbol represents the determination for an individual mouse. Horizontal lines represent the mean. The asterisks indicate p values <0.05 (*) or <0.001 (**). Statistical significance was determined by the Mann-Whitney U test.

To determine whether c1(96–100) mice have a generalized breach of B cell anergy that could contribute to the production of autoantibodies, we crossed anti-HEL Ig and sHEL transgenes onto the c1(96–100) background. Mice were aged up to 6 months and production of anti-HEL antibodies was contrasted with that of B6 mice. As shown in Fig 1A, c1(96–100) DTg mice produced significantly elevated levels of IgM^a^ anti-HEL antibodies as compared to B6 DTg mice, despite equivalent levels of total IgM^a^ in the two strains (B6 DTg = 0.11 ± 0.13 OD; c1(96–100) DTg = 0.13 ± 0.14 OD). Although the anti-HEL Ig transgene has been inserted outside of the Ig locus in this model and cannot undergo class-switch recombination, it is still possible to get expression of endogenous IgM^b^ heavy and light chains due to poor allelic exclusion. While there was minimal IgM^b^ anti-HEL antibody production in both strains, there were significantly elevated levels of IgG anti-HEL antibodies in both IgTg and DTg c1(96–100) as compared to corresponding B6 mice, which achieved quite high levels in some mice. As for IgM^a^, no significant differences between the total levels of IgM^b^ (B6 DTg = 0.40 ± .24 OD; c1(96–100) DTg = 0.71 ± 0.31 OD) or IgG (B6 DTg = 1.00 ± 0.09 OD; c1(96–100) DTg = 1.00 ± 0.10 OD) were observed between the B6 and c1(96–100) DTg strains. Comparable levels of IgG anti-HEL antibodies were seen both in Ig and DTg mice, indicating that these antibodies did not require the presence of HEL for their production and that T cell tolerance to HEL did not appear to significantly impair production of anti-HEL antibodies in c1(96–100) DTg mice. This finding suggests that these anti-HEL antibodies arose from IgM^b^ heavy and light chain pairings that retained specificity for HEL and that appear to have been activated to undergo class switching and differentiation to antibody-producing cells. Notably, production of IgG anti-ssDNA antibodies was retained or slightly increased in IgTg and DTg as compared to nTg c1(96–100) mice, despite the relative lack of B cells expressing endogenous heavy chains in these mice (Table 1).

**Table 1:** Comparison of splenic HEL^+^ and IgM^a+^ B cell subsets in B6 and c1(96–100) DTg mice.

*Cell Population (% of B220*^*+*^ *cells)*	*B6 (n = 6)*	*C1(96–100) (n = 18)*	*p value*
*HEL^+^*	*90.60±3.05*	*85.33±5.22*	***0.006***
*IgM^a-^HEL^-^*	*6.22±2.23*	*9.77±4.02*	***0.011***
*IgM^ahi^HEL^lo/-^*	*1.31±0.71*	*2.11±0.87*	***0.010***
*IgM^a+^IgM^b-^*	*88.71±4.24*	*86.78±3.79*	*0.095*
*IgM^a-^IgM^b+^*	*3.83±1.43*	*5.71±4.24*	***0.019***
*Igλ^+^*	*2.80±1.12*	*4.06±1.65*	***0.033***
*Igλ^+^IgM^a+^*	*1.10±0.38*	*1.34±0.58*	*0.410*

Cell populations were gated as shown in S1 Fig. Results shown are mean ± standard deviation. Significance levels were determined by the Mann-Whitney U test, with significant differences (p<0.05) indicated in bold.

Consistent with a breach of tolerance to HEL in c1(96–100) DTg mice, there were increased numbers of HEL-binding antibody-producing cells within the bridging channels, red pulp, and marginal zone of the spleen compared with their B6 counterparts (Fig 1B). Increased proportions of long-lived bone marrow plasma cells (Fig 1C) and splenic germinal center B cells (Fig 1D) were also seen in c1(96–100) DTg as compared to B6 DTg mice.

### c1(96–100) DTg B cells show altered function consistent with impaired anergy induction

Given the presence of anti-HEL antibodies in c1(96–100) DTg mice, we next sought to determine whether anergy induction was impaired. As shown in Table 1 (gating strategy shown in S1 Fig), over 85% of the B cells in both B6 and c1(96–100) DTg mice bound to HEL. There was a reduction of approximately 5% in the proportion of HEL-binding cells in c1(96–100) as compared to B6 DTg mice, which appeared to arise from increased proportions of B cells expressing endogenous heavy chains (IgM^b^ or IgG), or that had rearranged endogenous light chains. These endogenous chains appeared to have replaced transgenic heavy or light chains on the surface of the B cell resulting in a loss of specificity for HEL. These findings suggest that inhibition of endogenous heavy and light chain gene rearrangement is slightly impaired, or light chain editing slightly increased, in c1(96–100) mice.

**Fig 2:**
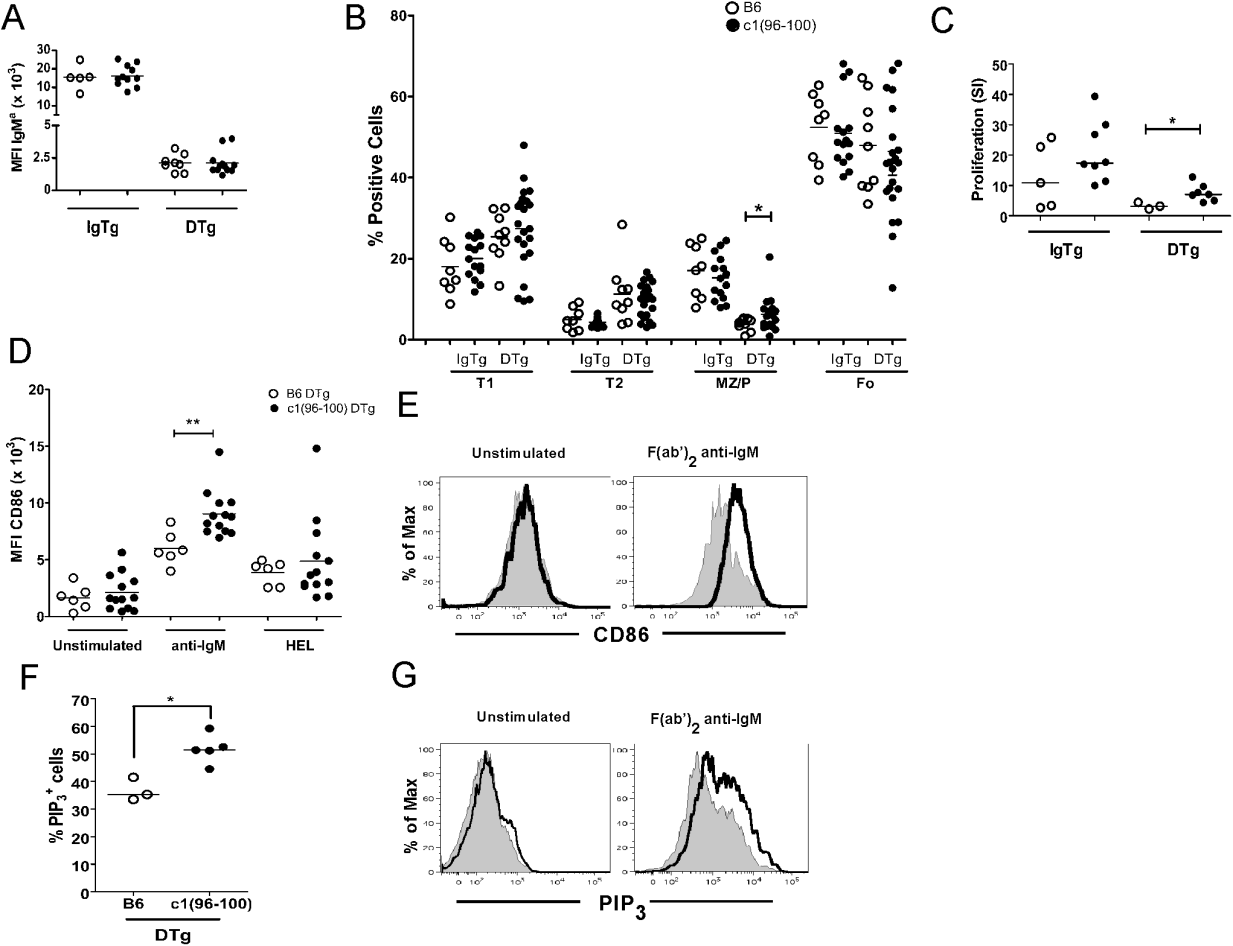
c1(96–100) DTg B cells show altered function consistent with impaired anergy induction. (A) Surface levels of IgM^a^ on splenic B (B220^+^) cells from 4-month-old B6 (open circles) and c1(96–100) (filled circles) IgTg or DTg mice were measured using flow cytometry (see S1 Fig for representative plot showing typical levels of IgM^a^ on B220^+^ cells). (B) Scatter plots showing the proportions of T1 (CD24^hi^CD21^-^), T2 (CD24^hi^, CD21^int^), follicular (CD24^lo^, CD21^+^; Fo) and marginal zone/precursor (CD24^int^, CD21^hi^; MZ/P) B cells in B6 (open circles) and c1(96–100) (filled circles) IgTg and DTg mice. Cells were gated as shown in S2A Fig. (C) Purified B cells from B6 (open circles) and c1(96–100) (filled circles) DTg mice were stimulated in vitro with HEL (0 or 100 ng/ml) together with a submitogenic concentration of LPS (50 ng/mL). B cell proliferation was measured by [^3^H]-thymidine incorporation at 36 hours by pulsing the cells overnight with 1 μCi/well. Uptake of [^3^H]-thymidine was quantified using a scintillation counter and expressed as the mean CPM of triplicate wells. The stimulation index (SI), the ratio of stimulated (HEL+LPS) to un-stimulated (LPS only) cells, was calculated for each experiment. Results shown are from three independent experiments. (D) Splenocytes from B6 (open circles) or c1(96–100) DTg (filled circles) mice were stimulated in media alone, with anti-IgM F(ab’)_2_ (10 μg/ml), or with HEL (100 ng/ml) for 18 hours. Cultured cells were stained with anti-IgM^a^, -CD86, and -B220, and analyzed by flow cytometry. CD86 levels were gated on IgM^a+^B220^+^ cells, or for the anti-IgM stimulation for IgTg mice on all B220^+^ cells, as shown in S2B Fig. (E) Representative histograms showing the CD86 expression on B6 DTg (thin line, filled histograms) vs c1(96–100) DTg (bold line) IgM^a+^B220^+^ B cells for unstimulated and stimulated conditions. (F) To quantify phosphatidylinositol 3,4,5,-triphosphate (PIP_3_) production, splenocytes were stimulated with anti-IgM F(ab)’_2_ Ab for 5 min, fixed with 1% paraformaldehyde, stained with anti-B220, permeabilized, and then stained intracellularly with an anti-PIP_3_ Ab. Scatter plots show the percentage of PIP_3_^+^ cells from DTg mice from B6 (open circles) or c1 (96–100) (filled circles), calculated as the expression of PIP_3_ on stimulated B220^+^ B cells above the media control. (G) Representative histograms showing PIP_3_ expression in B6 DTg (thin line) vs c1(96–100) DTg (bold line) B220^+^ B cells for unstimulated and stimulation conditions. For scatterplots, each symbol represents the determination for an individual mouse. Horizontal lines represent the mean. The asterisks indicate p values <0.05 (*) or <0.001 (**). Statistical significance was determined by Mann-Whitney U test.

Consistent with an anergic phenotype [15–17], both B6 and c1(96–100) B cells demonstrated equivalent down-regulation of IgM^a^ in DTg as compared to IgTg mice (Fig 2A). Normally, anergic B cells are excluded from the marginal zone and demonstrate impaired selection into the mature follicular B cell compartment [32–34]. Consistent with this, there were increased proportions of T1/T2 B cells and decreased proportions of follicular and marginal zone cells in DTg as compared to IgTg mice for both mouse strains (Fig 2B, gating strategy shown in S2A Fig). Comparison of B6 and c1(96–100) DTg mice revealed similar proportions of splenic B cells in all of these subsets, with the exception of a slight but significant increase in the proportion of marginal zone/marginal zone precursor cells in c1(96–100) DTg mice (B6 = 3.8 ± 1.5, c1(96–100) = 6.3 ± 3.8, p = 0.035).

Anergic B cells demonstrate a number of functional changes including impaired proliferation and reduced up-regulation of CD86 in response to antigen engagement [35–37]. As seen in Fig 2C, DTg B cells from both strains showed less proliferation than IgTg B cells following stimulation with HEL and submitogenic LPS. However, there was a significant increase in the proliferation of c1(96–100) as compared to B6 DTg B cells, suggesting that c1(96–100) DTg B cells are slightly less anergic than their B6 counterparts. Consistent with this possibility, c1(96–100) DTg B cells demonstrated enhanced up-regulation of CD86 compared to B6 DTg B cells following stimulation with anti-IgM, but not HEL (Fig 2D). This increase was not due simply to an increased proportion of edited cells, because there was a greater shift in the levels of CD86 expression for all IgM^a+^ c1(96–100) DTg B cells as compared to B6 DTg B cells following stimulation (Fig 2E).

Previous experiments have shown that impaired responsiveness in DTg HEL-specific B cells is associated with suppression of phosphatidylinositol 3,4,5-triphosphate (PIP_3_) production [38]. Therefore, to further explore whether impairment of this signaling pathway is altered in c1(96–100) DTg mice B cells, we assessed intracellular PIP_3_ levels following BCR cross-linking with anti-IgM. As shown in Fig 2F, PIP_3_ production was increased in c1(96–100) as compared with B6 DTg B cells following activation, and similar to what was seen for CD86 expression, this was observed for all cells (Fig 2G). Taken together, the data indicate that c1(96–100) have mildly impaired anergy induction.

### Antigen-engaged c1(96–100) B cells demonstrate enhanced survival

**Fig 3:**
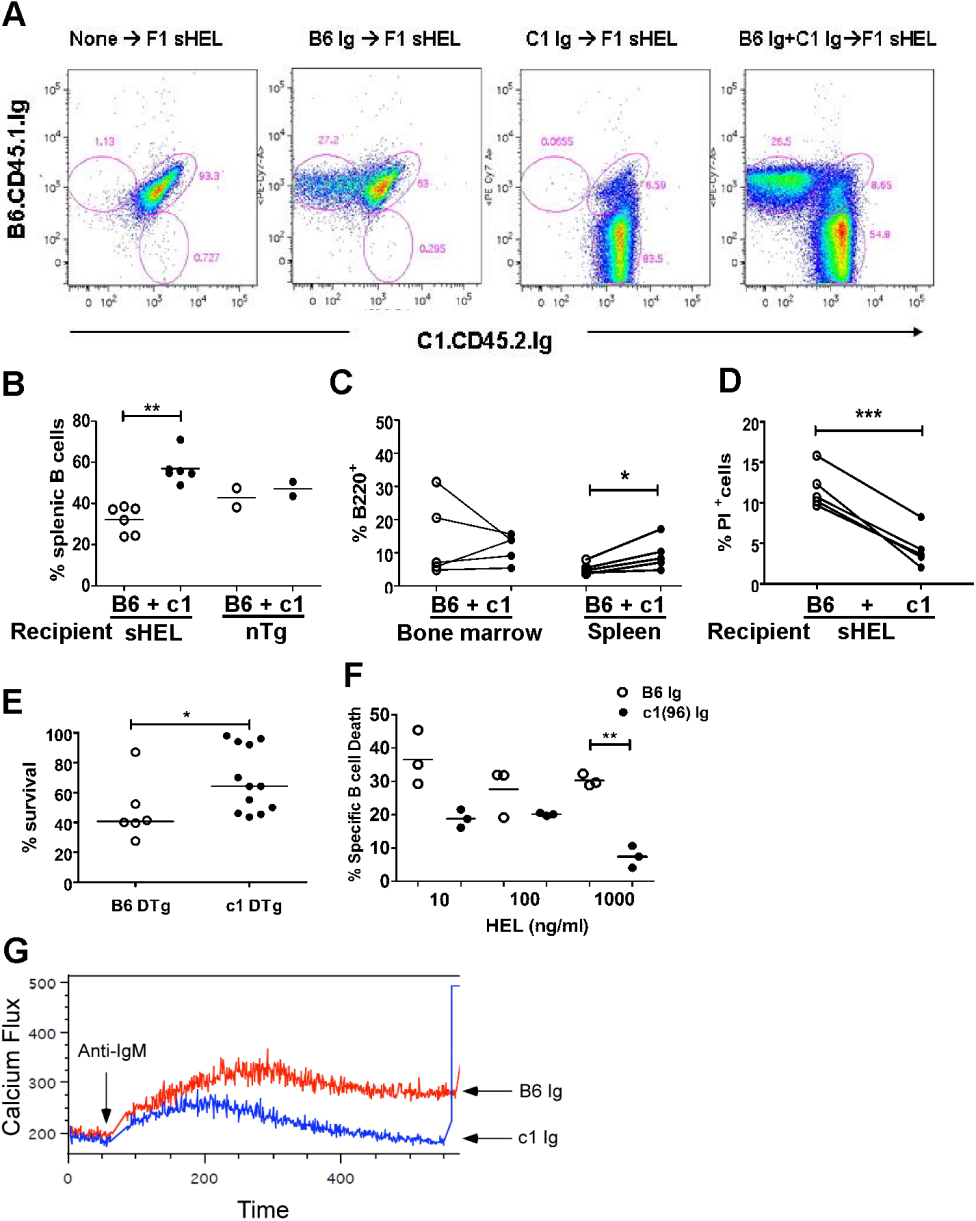
Antigen-engaged c1 congenic B cells demonstrate enhanced survival. (A) Flow plots showing reconstituted splenic B (B220^+^) cells in F1 sHEL mice. Irradiated F1 sHEL or nTg recipients were reconstituted with bone marrow cells from B6.CD45.1.IgTg mice, c1(96–100).CD45.2.IgTg mice, or both. (B) Scatter plots showing the proportion of B220^+^IgM^a+^ splenocytes that are CD45.1^+^ (B6.IgTg; open circles) or CD45.2^+^ (c1(96–100).IgTg; closed circles) in sHEL or nTg recipient mice reconstituted with a mixture of B6.CD45.1 and c1(96–100) IgTg bone marrow (MC). (C) Proportion of cells within the bone marrow or spleen that were CD45.1^+^B220^+^ (B6) or CD45.2^+^B220^+^ (c1(96–100)) cells in sHEL MC recipient mice. Each paired data set shows a comparison between the proportion of CD45.1^+^B6.IgTg B cells (open circles) and CD45.2^+^ c1(96–100).IgTg B cells (closed circles) within an individual mouse. (D) In MC mice, B cell death was determined by gating on CD45.1^+^ (B6) or CD45.2^+^ (c1(96–100) B220^+^ cells and then quantifying the proportion of PI^+^ cells for each population. Lines connect data for c1(96–100) (filled circles) and B6 (open circles) B cells within an individual sHEL MC recipient, as in (C). (E) Purified B cells from the spleens of B6 (open circles) or c1(96–100) (filled circles) DTg mice were stimulated in media alone or with HEL (100 ng/ml) for 18 hours. Cultured cells were stained with anti-IgM^a^ and -B220, and analyzed by flow cytometry. Scatter plots show % B cell survival, expressed as the percentage of B220^+^ cells surviving in HEL divided by the percentage surviving in media alone. (F) Bone marrow cells from B6.IgTg (open circles) or c1(96–100).IgTg (filled circles) mice were cultured in the presence of IL-7 for 5 days to produce naïve immature B cells. Cultured cells were stimulated in media alone or with HEL (at various concentrations) for 18 hours. Cells were then stained with B220 and PI, and analyzed by flow cytometry. Scatter plots show the percentage of PI^+^ B220^+^ cells as a measure of B cell death. (G) Calcium mobilization in naïve immature IgTg B cells from B6 (red) and c1(96–100) (blue) mice was measured by flow cytometry. Cultured immature B cells were labeled with Indo-1 and cross-linked with anti-IgM Ab. For panels B-F, each symbol represents the determination for an individual mouse. Horizontal lines represent the mean. The asterisks indicate p values <0.05 (*) or <0.001 (**). Statistical analyses were performed using Mann-Whitney *U* tests for (B), (E) and (F), or pair-study non-parametric tests for (C) and (D).

To further explore the immune mechanisms leading to the breach of B cell tolerance to HEL in c1(96–100) DTg mice and to establish the role of altered B cell function in this process, we generated hematopoietic chimeric mice with a mixture of B6 and c1(96–100) IgTg bone marrow. To this end, B6.CD45.1 mice were bred with c1(96–100).sHEL mice to produce sHEL and non-Tg (B6.CD45.1 x c1(96–100))F1 recipient mice, which were then lethally irradiated at 8 wks of age and reconstituted with a 1:1 mixture of B6.CD45.1 IgTg and c1(96–100) IgTg bone marrow. Fig 3A shows representative flow cytometry plots for sHEL F1 mice reconstituted with various bone marrow combinations. Under the irradiation conditions utilized, there was some residual survival of F1 hematopoietic cells in the spleen but not the bone marrow. These could be easily discriminated from the B6 and c1(96–100) cells by their dual staining with anti-CD45.1 and -CD45.2 antibodies. In non-Tg recipient mice reconstituted with both B6.CD45.1 and c1(96–100) IgTg bone marrow, there were equivalent proportions of IgM^a+^ B6.CD45.1 and c1(96–100) B cells in the spleen after reconstitution. In contrast, the proportion of IgM^a+^ B6.CD45.1 B cells was significantly reduced as compared to IgM^a+^ c1(96–100) B cells in the spleens of sHEL F1 recipient mice (Fig 3B). This reduction was not the result of impaired reconstitution of B6.CD45.1 B cells in sHEL F1 recipient mice, because the proportion of B6.CD45.1 bone marrow B cells was not significantly different from that of c1(96–100) bone marrow B cells (Fig 3C). This finding suggested that that there may be enhanced survival of chronically antigen-engaged HEL-specific c1(96–100) peripheral B cells. Consistent with this possibility, there were decreased proportions of PI^+^ c1(96–100) as compared to B6.CD45.1 B cells in the spleens of sHEL F1 recipient mice (Fig 3D).

In agreement with the concept that c1(96–100) splenic B cells show enhanced survival as compared to B cells from B6 mice, splenic B cells from c1(96–100) congenic DTg mice demonstrated significantly increased survival following stimulation with HEL overnight, as compared to their B6 counterparts (Fig 3E).To further explore the possibility that apoptosis of antigen-engaged B cells is reduced in c1(96–100) congenic mice, bone marrow cells were isolated from B6 and c1(96–100) IgTg mice and cultured in-vitro with IL-7 for 5 days to produce predominantly immature B cells. The cells were then cross-linked with HEL and apoptosis was quantified by PI exclusion. As previously observed for their mature counterparts, c1(96–100) immature bone marrow-derived B cells demonstrated significantly decreased cell death compared to their B6 counterparts (Fig 3F). Since the extent of apoptosis is closely related to the strength of the B cell receptor signal for immature B cells [39,40], calcium mobilization was examined following IgM receptor cross-linking. As shown in Fig 3G, immature IgTg c1(96–100) B cells had significantly attenuated signaling as compared to B6 B cells, suggesting that the reduced apoptosis in these cells arises at least in part from attenuated BCR signaling.

### Enhanced TFH differentiation and an increase in long-lived plasma cells contribute to the breach of anergy in c1(96–100) mice

**Fig 4:**
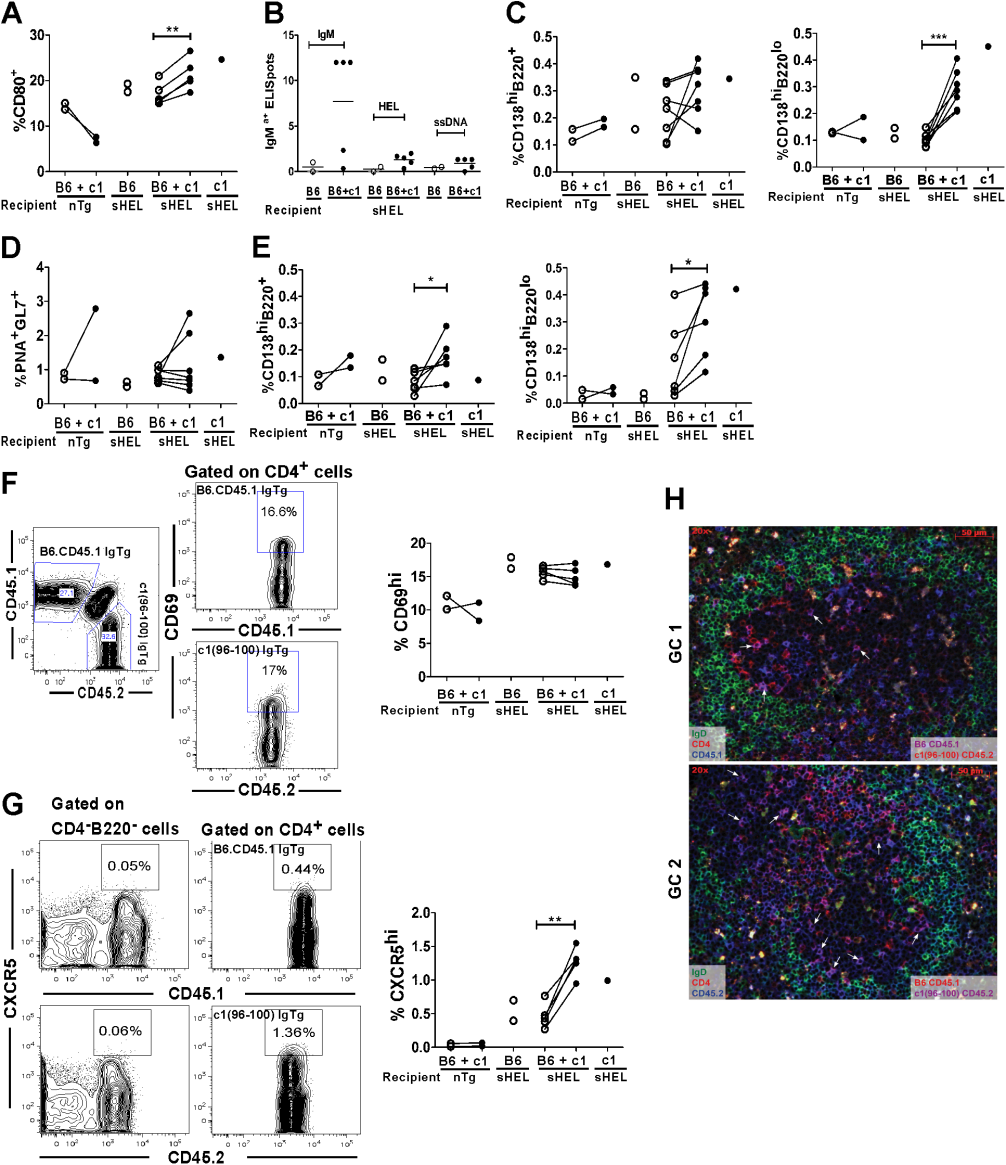
Comparison of various B and T cell populations in mixed hematopoietic chimeric sHEL recipient mice. (A) Reconstituted B cells expressing the activation marker CD80 were examined by flow cytometry. Scatter plots show a comparison of the proportion of CD80^+^ cells within the B220^+^ subset of the B6.CD45.1.IgTg (open circles) and c1(96–100).IgTg (closed circles) cell populations for the indicated reconstituted mice, gated as shown in (F). (B) The number of IgM^a+^ anti-IgM, anti-HEL, or anti-ssDNA Ab-producing splenocytes from sHEL recipient mice was quantitated by ELISpot, where the y-axis value corresponds to the number of spots per half million cells. Each circle represents the determination from a single recipient mouse. Open circles indicate mice reconstituted with cells of B6.CD45.1.IgTg origin only and filled circles indicate mice that received both B6.CD45.1IgTg and c1(96–100).IgTg cells. Horizontal lines indicate the mean for each group. (C) Flow cytometric analysis of plasmablasts (CD138^hi^ B220^+^) and plasma cells (CD138^hi^B220^lo/-^) in MC and control mice. Scatter plots show the proportion of cells in the B6.CD45.1.IgTg (open circles) and c1(96–100).IgTg (closed circles) populations within the spleens of nTg or sHEL recipient mice. (D) Scatter plots show the proportions of germinal center cells within the B220^+^ subset of the B6.45.1.IgTg (open circles) and c1(96–100).IgTg (closed circles) populations of the spleens of individual nTg or sHEL F1 recipient mice. Live (PI-excluding) B6.CD45.1 or c1(96–100) were first gated as shown in (F) and then B cells gated as B220^+^. The PNA^+^GL7^hi^ GC subset was then determined, as shown in Fig 1D. (E) Scatter plots comparing the proportions of B6.CD45.1.IgTg (open circles) and c1(96–100).IgTg (closed circles) early (CD138^hi^B220^+^) and long-lived plasma cells (CD138^hi^B220^lo/-^) in the bone marrow of nTg or sHEL recipient mice. (F) Left flow plot shows regions used to gate the B6.CD45.1.IgTg and c1(96–100).CD45.2.IgTg cells in reconstituted mice. Right flow plots show representative gating of the early activation marker CD69 on CD4^+^ T cells gated on CD45.1^+^ (top) or CD45.2^+^ (bottom) cells. CD69^hi^ gates were set based on the background levels of CD69 on CD45.1^+^ or CD45.2^+^ non-T, non-B cells. Scatter plots show the proportion of CD69^hi^ cells within the CD4^+^ T cell subset of the B6.CD45.1.IgTg (open circles) and c1(96–100).IgTg (closed circles) populations in the spleens of nTg or sHEL recipient mice. (G) Flow plots showing the regions used to gate a T cell subset enriched for TFH cells (CXCR5^hi^CD4^+^). Left set of plots are gated on CD4^-^B220^-^ cells and show how the CXCR5^hi^ region was determined together with the background levels of staining in this population in the CD45.1^+^ (top) and CD45.2^+^ (bottom) cell subsets. Right set of plots are gated upon B6.CD45.1.IgTg (Top) and c1(96–100).IgTg (bottom) CD4^+^ cells. Scatter plots compare the proportions CXCR5^hi^cells within the CD4^+^ subset of the B6.CD45.1.IgTg (open circles) and c1(96–100).IgTg (closed circles) populations in the spleens of individual nTg or sHEL recipient mice reconstituted with various bone marrow combinations. (H) Immunofluorescent imaging of TFH cells in the GC of two representative MC mice. Spleen sections have been stained with biotinylated-anti-CD45.1 or -anti-CD45.2 followed by streptavidin-AMCA (blue), together with FITC anti-IgD (green) and PE-anti-CD4 (red). TFH cells of either B6 CD45.1 (top image) or c1(96–100) CD45.2 (bottom image) origin are indicated by the arrows. Magnification x20. For scatterplots, the asterisks indicate p values <0.05 (*) or <0.001 (**). Statistical analyses were performed using a pair-study non-parametric test.

In addition to enhanced survival, c1(96–100) B cells in the spleens of sHEL mixed chimeric (MC) mice had elevated levels of CD80 on their cell surface as compared to B6.CD45.1 B cells (Fig 4A). Since sHEL F1 mice reconstituted with both B6.CD45.1 and c1(96–100) IgTg bone marrow had a trend to increased numbers of HEL-specific ELISpots as compared to mice reconstituted with B6 IgTg bone marrow alone (Fig 4B), we expected that the predominant cells contributing to this breach of tolerance would be the c1(96–100) B cells. However, this was not the case. Equivalent proportions of B6.CD45.1 and c1(96–100) plasmablasts (CD138^hi^B220^+^) and GC B cells (B220^+^PNA^hi^GL7^hi^) were seen in the spleens of sHEL F1 recipient mice, as assessed by flow cytometry (Fig 4C & 4D). Similar findings were observed when HEL-specific antibody-producing cells in the spleen were examined by immunofluorescence microscopy (S3 Fig), suggesting that an intrinsic B cell anergy defect is not required for differentiation of self-reactive HEL-specific cells to plasmablasts or recruitment into GC in the MC environment. In contrast, the proportions of c1(96–100) splenic plasma cells and bone marrow early and long-lived plasma cells were increased as compared to their B6.CD45.1 counterparts in sHEL MC mice (Fig 4C & 4E). For splenic plasma cells and bone marrow early plasma cells these findings recapitulated what was seen in the corresponding DTg mouse strains and in sHEL F1 mice reconstituted with B6.CD45.1 or c1(96–100) bone marrow alone, suggesting that these changes arise from an intrinsic c1(96–100) B cell functional defect. However, the proportion of B6.CD45.1 bone marrow long-lived plasma cells seen in the majority of sHEL MC was increased as compared to those seen in sHEL F1 mice reconstituted with B6 bone marrow alone, reinforcing the concept that MC environment, most likely through the presence of T cell help, also contributes to the breach of tolerance in these mice.

To further explore the role of T cells in MC mice, we examined whether there were differences in the activation of c1(96–100) as compared to B6.CD45.1 T cells that could account for the overall trend to increased proportions of splenic antibody-producing and bone marrow plasma cells in sHEL MC recipient mice as compared to those reconstituted with B6 bone marrow alone. There were equivalent proportions of recently activated (CD69^hi^CD4^+^) B6.CD45.1 and c1(96–100) T cells in the spleens of sHEL MC recipient mice (Fig 4F). TFH cells are known to be important for the development of GC B cells, production of anti-DNA Abs, and generation of long-lived bone marrow plasma cells [29,37,41]. Although stains permitting unequivocal identification of TFH cells (CD4^+^CD44^hi^CXCR5^hi^PD-1^hi^) were not performed as part of this experiment, we and others have previously shown that the CD4^+^CXCR5^hi^ population is largely PD-1^hi^ and CD44^hi^, indicating that this stain can be used to examine a population that is enriched for TFH cells [29,42]. As shown in Fig 4G, the proportion of c1(96–100) CD4^+^ cells that were CXCR5^hi^ was significantly elevated as compared to their B6 counterparts. Furthermore, in sHEL MC recipient mice, the proportion of B6 or c1(96–100) cells within this cell subset appeared to recapitulate that seen in corresponding sHEL recipient mice reconstituted with B6 or c1(96–100) alone. Notably, the enhanced CXCR5^hi^CD4^+^ T cell differentiation for c1(96–100) cells was not seen in nTg MC recipient mice, suggesting that it is dependent upon the presentation of sHEL by IgTg B cells. To further investigate whether c1(96–100) TFH were increased as compared to B6.CD45.1 TFH within the GC of sHEL MC mice, spleen sections were stained to identify GC, and B6.CD45.1 or c1(96–100) TFH were identified as CD4^+^ CD45.1^+^ or CD45.2^+^ cells, respectively, within the GC (Fig 4H). As suggested by the flow cytometry results, increased numbers of c1(96–100), as compared to B6, CD4^+^ cells were seen in the GC of sHEL MC mice. Taken together these findings suggest that c1(96–100) T cells have an enhanced ability to differentiate to TFH cells in the context of self-tolerance and that this contributes to the breach of B cell anergy in c19(6–100) mice.

### Increased differentiation of TFH and pro-inflammatory T cell subsets in c1(70–100) DTg mice is associated with an enhanced breach of B cell tolerance to HEL

**Fig 5:**
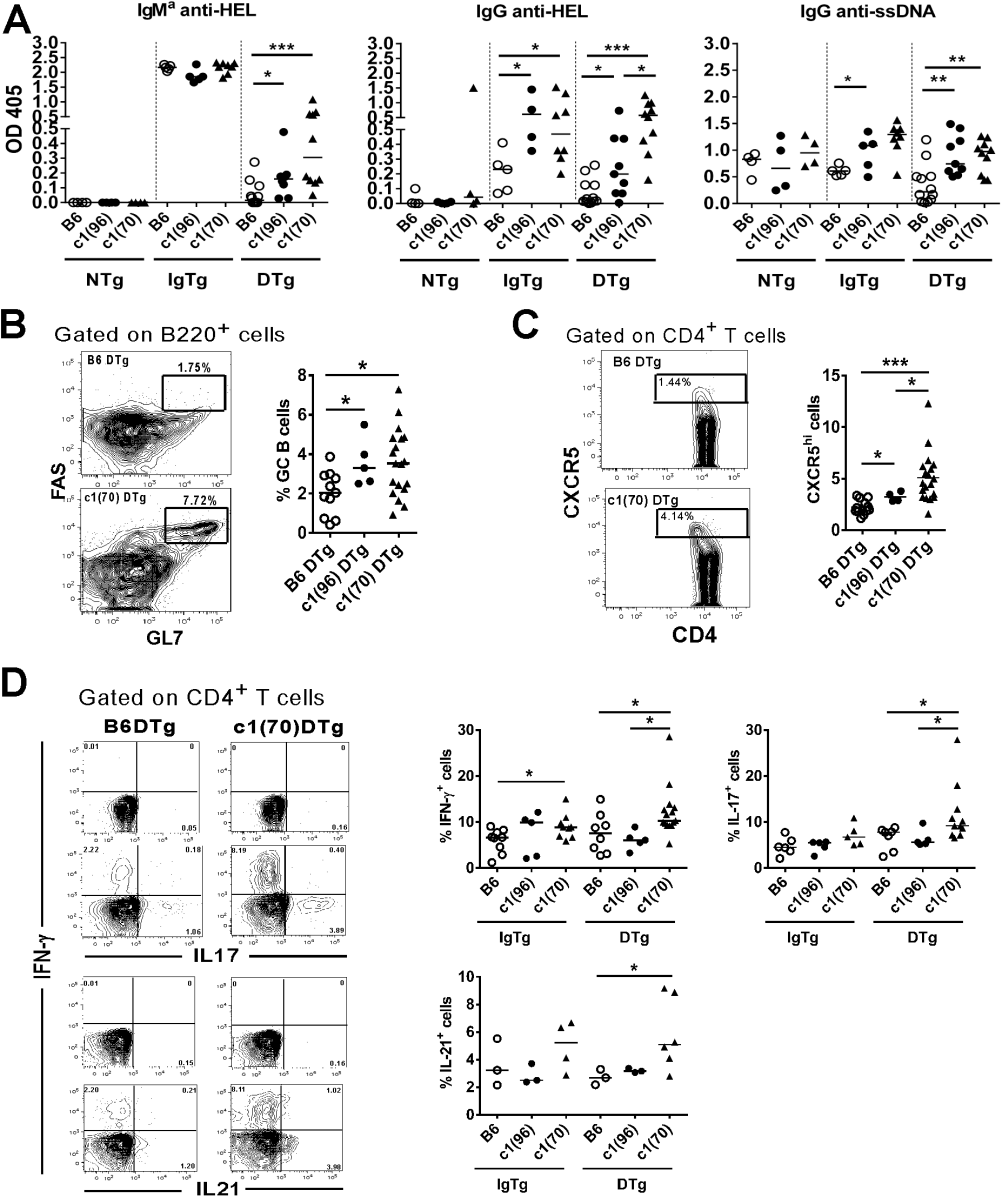
c1(70–100) DTg mice have an enhanced breach of B cell tolerance. (A) Scatter plots showing serum levels of IgM^a^ anti-HEL, IgG anti-HEL and IgG anti-ssDNA Abs from 8 week-old to 4 month-old B6 (open circles), c1 (96–100) (filled circles) and c1(70–100) (filled triangles) mice, as measured by ELISA. (B) Shown on the left are flow plots gated upon B220^+^ cells indicating the regions used to gate GC B cells. Scatter plots show the proportion of GC cells within the B220^+^ subset of B6 DTg (open circles), c1(96–100) DTg (filled circles) or c1(70–100) DTg (filled triangles) mice. (C) Flow plots gated on CD4^+^ T cells showing the regions used to gated CXCR5^hi^ cells (regions were established as outlined in Fig 4G). Scatter plots show the proportion of these cells within the CD4+ T cell subset in B6 DTg (open circles), c1(96–100) DTg (filled circles), or c1(70–100) DTg (filled triangles) mice. (D) Representative flow cytometry contour plots showing the gating used to identify the proportion of IFN-γ-, IL-17-, and IL-21-producing CD3^+^CD4^+^ T cells in c1(70–100) DTg mice. The quadrants used to define positively and negatively stained cells are indicated. For each condition, the upper plots show unstimulated cells and the lower plots show cells stimulated with PMA and ionomycin. Scatter plots showing the proportion of cytokine-producing T cells in B6 (open circles), c1(96–100) (filled circles) and c1(70–100) (filled triangles) mice. Each symbol represents an individual mouse. Horizontal lines represent the mean. The asterisks indicate p values <0.05 (*) or <0.001 (**). Statistical analyses were performed using a Mann-Whitney *U* test.

Given the potential role of T cells in the production of anti-HEL antibodies in c1(96–100) DTg mice, we were interested in determining whether lupus susceptibility loci that impact predominantly on T cell function would augment autoantibody production in this system. We have previously shown that c1(70–100) congenic mice with a longer NZB chromosome 1 interval encompassing the c1(96–100) interval have T and dendritic cell defects that lead to increased proportions of TH1, TH17, and TFH cells as compared to c1(96–100) mice [29]. To determine the impact of these additional defects on HEL-specific tolerance, we crossed anti-HEL Ig and sHEL transgenes onto the c1(70–100) background. Functional analysis of immature IgTg c1(70–100) B cells revealed similar changes to those observed for c1(96–100) mice (S4 Fig). Consistent with the lack of evidence for additional B cell defects in c1(70–100) mice there were no significant differences between c1(96–100) and c1(70–100) DTg mice in any of the B cell subsets examined (S1 Table). As predicted, both c1(70–100) IgTg and DTg mice produced significantly elevated levels of IgG anti-HEL antibodies, and had a trend to increased levels of IgM^a^ anti-HEL antibodies, as compared with their c1(96–100) counterparts (Fig 5A). These results affirm that in the presence of the T cell abnormalities in the longer NZB chromosome 1 interval, the breach of B cell anergy and subsequent autoantibody production are enhanced.

Since our previous work showed that c1(70–100) mice have larger GCs and an expansion of TFH cells as compared to c1(96–100) mice [29], we examined to what extent these altered GC processes impact on the breach of tolerance in anergic B cells. Flow cytometric analysis of splenocytes from c1(70–100) and c1(96–100) DTg mice revealed equivalently increased proportions of c1(70–100) and c1(96–100) DTg GC B cells as compared to B6 DTg mice (Fig 5B). Despite the equivalent proportions of GC B cells in the two mouse strains, there was a significantly higher percentage of CXCR5^+^ cells within the CD4^+^ T cell subset of c1(70–100) DTg mice compared with their c1(96–100) DTg counterparts (Fig 5C) and the proportions of these cells in both these mouse strains was higher than that seen in B6 DTg mice. Since we have previously shown that c1(70–100) mice demonstrate enhanced production of a variety of T cell-derived pro-inflammatory cytokines [29], we investigated whether increased cytokine production by CD4^+^ T cells could be partly responsible for the observed differences. Purified CD4^+^ T cells from IgTg or DTg mice were activated with PMA and ionomycin, and intracellular cytokine production was analyzed by flow cytometry (Fig 5D). Increased proportions of IFN- γ-, IL-17- and IL-21-producing T cells were seen in c1(70–100) DTg as compared to B6 DTg mice, and for IFN-γ and IL-17 were statistically significant increased as compared to c1(96–100) DTg mice. Overall, these findings provide support for the role of T cells in the breach of B cell anergy in c1 congenic mice.

## Discussion

Studies in lupus-prone mice have revealed numerous B and T cell tolerance defects that contribute to autoantibody production [13,23,29,43]; however, the manner in which these tolerance defects reinforce one another, and the critical point at which the balance tips to autoimmunity, remains unclear. In this study, we show that mice with the 96–100 cM interval from NZB chromosome 1 breach anergy to the neo-self antigen HEL, with significantly higher levels of anti-HEL Abs and antibody-producing cells compared with their non-autoimmune B6 counterparts. This breach is recapitulated in hematopoietic chimeric mice with a mixture of B6 IgTg and c1(96–100) IgTg B cells. Surprisingly, despite impaired anergy induction and apoptosis of c1(96–100) IgTg B cells in these mice, the predominant abnormality that appears to promote this breach is the enhanced differentiation of c1(96–100) T cells to TFH cells, which is compounded by a B cell intrinsic accumulation of long-lived plasma cells. In further support of the importance of T cells in this breach, production of IgG anti-HEL antibodies was significantly increased in c1(70–100) DTg mice, which have increased proportions of TFH cells and IFN-γ-, IL-21- and IL-17-producing T cells as compared to c1(96–100) DTg mice.

Although the NZM2410 *Sle1b* and *Sle1* loci are NZW- and not NZB- derived, a breach of B cell anergy to HEL analogous to what we have observed in the c1(96–100) and c1(70–100) mice, respectively, has been reported for these strains [20]. Similar to the NZB c1 DTg mice, B6.*Sle1*.HEL^Ig^.sHEL mice had increased production of IgM^a+^ anti-HEL antibodies compared with B6.HEL^Ig^.sHEL controls. However, in contrast with our findings, B6.*Sle1*.HEL^Ig^.sHEL mice did not display elevated levels of IgG anti-HEL or -DNA antibodies. Furthermore, while c1(96–100) DTg mice had increased proportions of IgM^b+^ and Igλ^+^ B cells in the spleen and bone marrow, indicative of increased receptor editing and/or impaired allelic exclusion of HEL-specific (IgM^a+^) to endogenous BCRs, B6.*Sle1*.HEL^Ig^.sHEL mice showed the opposite, with significantly increased proportions of IgM^a+^ splenic B cells. The lack of IgG antibodies, coupled with a reduced proportion of activated CD4^+^ T cells in the spleens of B6.*Sle1*.HEL^Ig^.sHEL mice, led the authors to conclude that the breach of anergy seen in these mice was due to B and not T cell defects. The reason for the discrepancies between our findings and those of this group are unclear. Since the role of TFH cells had not yet been appreciated at the time of this publication and the T cell differences observed in our mice were predominantly in this cell subset, it is possible that the contribution of T cells was missed. Alternatively, the differences observed between *Sle1* and NZB c1 mice, which have been previously reported to share the same *Slam* locus, may arise from genetic polymorphisms between these two mouse strains. Consistent with this possibility, recent whole genome sequencing of NZB and NZW mice (http://www.sanger.ac.uk/) has revealed numerous polymorphisms between these strains localized within the *Slam* locus. These genetic changes could directly impact on T cell function or alternatively indirectly impact on the ability of B cells to activate or be activated by T cells. In connection with the later possibility, the increased production of anti-HEL IgG antibodies observed in our mice is derived from B cells that have endogenous heavy chains, a subset that was reported to be significantly reduced in *Sle1* mice. Thus, despite the similarity in their genetic loci [19], there may be fundamental differences between NZM2410 and NZB mice that change how and when tolerance checkpoints are breached, leading to distinct B cell repertoires and autoantibody production in these strains.

In agreement with previous observations in the *Sle1* model [20], immature B cells demonstrated attenuated calcium mobilization in c1(96–100) mice. As the strength of B cell receptor signaling plays an important role in tolerance induction [39], it is probable that the impaired immature B cell apoptosis, anergy induction, and allelic exclusion observed in these mice results at least in part from this signaling abnormality. This signaling may also be responsible for the increased levels of CD80 on c1(96–100).IgTg as compared to B6.CD45.1.IgTg splenic B cells in MC sHEL mice, as the impaired anergy induction in c1(96–100) B cells is associated with an increased ability to up-regulate costimulatory molecules in response to antigen engagement. Surprisingly, despite the impaired anergy induction in c1(96–100) B cells, these cells were equivalently recruited into germinal centers and the plasmablast compartment as compared to B6.IgTg B cells in MC sHEL mice. These findings suggest that once self-reactive T cells are activated, a B cell anergy defect is not required for these processes, and raise the possibility that the previously observed differences in recruitment of c1 as compared to B6 B cells into germinal centers in wild type MC mice resulted from differences in the B cell repertoire (perhaps related to the attenuated immature B cell signaling) rather than defective anergy induction, as we had previously proposed [13].

Whether the B cell anergy defect is required for activation of self-reactive T cells is currently unclear. Previous work has shown that the inability of anergic HEL-reactive B cells to up-regulate costimulatory molecules plays a critical role in preventing activation of HEL-reactive T cells in DTg mice and that constitutive expression of CD86 on anergic B cells is sufficient to breach tolerance in this model [34,36]. It is therefore possible that the enhanced upregulation of CD80 by c1(96–100).IgTg B cells in MC sHEL mice acts to promote activation and differentiation of HEL-reactive TFH cells. However, comparison of TFH differentiation in sHEL F1 mice reconstituted with B6 bone marrow alone or in combination with c1(96–100) bone morrow revealed equivalent differentiation of B6 TFH cells in the presence or absence of c1(96–100) B cells. Consequently, if increased CD80 expression by c1(96–100) B cells is required to induce activation of tolerance-breaching TFH cells, it must act in tandem with the c1(96–100) T cell functional abnormality. Such a model is consistent with our previous work showing that CD4^+^ T cells are necessary for anti-HEL antibody production in NZB DTg mice and that the abnormal survival, proliferation, and germinal center entry of NZB DTg B cells [23] following adoptive transfer into sHEL recipient mice requires both an intrinsic B cell functional defect and the presence of CD4^+^ T cells.

There is a growing body of work outlining the importance of germinal center tolerance mechanisms in the maintenance of B cell tolerance and the role of the *Slam* family in these GC tolerance checkpoints [25,26]. Consistent with a potential role for these molecules in c1 congenic mice, we have previously demonstrated the existence of increased germinal center responses in c1(96–100) and c1(70–100) wild type mice [29]. However, the fate of anergic B cells in the context of these altered germinal center responses remains unknown. It has been unclear whether this abnormal response arises predominantly from abnormal function of the B cell, T cell or a combination thereof. In this study, we show that the breach of B cell anergy in both c1(96–100) and c1(70–100) DTg mice is associated with high levels of IgG anti-HEL antibody production, significant increases in the proportion of germinal center B cells, and a qualitative increase in the overall numbers of GC in the spleen, strongly arguing that disturbed germinal center tolerance mechanisms contribute to the breach of tolerance in these mice. Notably, in F1 sHEL MC mice, both B6.CD45.1 and c1(96–100) B cells were found in similar proportions in germinal centers, suggesting that the altered germinal center tolerance arises predominantly from the abnormal activation of TFH cells in these mice. These findings are at odds with those reported for *Sle1* mice, where their B cells were shown to have enhanced survival in the GC [26]. Furthermore, despite previous reports of *Slam*-mediated T cell defects that can drive the breach of tolerance [21,27,44], GC formation and TFH expansion in *Sle1b* mice appeared to be caused solely by the B cell intrinsic defects [25]. The reasons for the differences between c1 congenic and *Sle1b* mice are currently unknown, but could arise from genetic polymorphisms or differences in the antigen systems being examined.

In contrast to the lack of differences between germinal center and plasmablast recruitment in sHEL MC mice, the proportion of c1(96–100) long-lived spleen and bone marrow plasma cells was increased as compared to B6 cells. This finding suggests that c1(96–100) mice have an intrinsic B cell functional defect that affects post-germinal center differentiation of B cells to long-lived plasma cells and/or the survival of these cells. While it is tempting to speculate that this results from polymorphisms in the *Slam* locus, the *Fcgr2b* locus is also located in the c1(96–100) interval [14]. Previous work indicates that NZB mice have decreased expression of Fcgr2b on the surface of their plasma cells and that this enhances the survival of these cells, potentially leading to increased autoantibody production [45].

Although GCs are important for the production of IgG autoantibodies, several lupus-prone mouse models including RF IgG2a, MRL.Fas^lpr^ (anti-DNA) and BAFF-Tg have a marked predominance of short-lived, splenic plasmablasts [46–48], which raises the question of how much of the breach of tolerance is mediated through GC responses as compared to extrafollicular activation. While we cannot exclude a potential role for extrafollicular activation in our model, our findings suggest that the breach of tolerance observed is predominantly T cell-dependent, since B6.CD45.1 B cells were only capable of differentiating to splenic anti-HEL antibody-producing cells in F1 sHEL recipient mice when c1(96–100) T cells were present.

In summary, our results definitively show that c1(96–100) and c1(70–100) lupus-prone mice have intrinsic defects in B cell function that are present early in B cell development and that lead to an impaired anergy induction. Once in the periphery, this anergy defect does not appear to contribute directly to increased recruitment to germinal centers. Instead, these anergic B cells may facilitate the priming and activation of antigen-specific T cells in the follicle, which then play a vital role at various extrafollicular and germinal center checkpoints to drive plasma cell differentiation and autoantibody production.

## Supporting information

S1 Fig Flow cytometry plots showing the regions used to gate various B cell populations. Splenocytes were stained as outlined in the materials and methods, and dead cells excluded by PI staining. Labels at the top of each cluster of graphs indicated the gated cells. Boxes in each plot show the regions used to identify various populations of B cells (corresponding to those in Table 1) with the specific populations being indicated on the plots for the DTg splenocytes. Numbers in the regions indicate the percentage of each population within the gated cells.
(TIF)

S2 Fig Flow cytometry plots showing regions used for gating in Fig 2. (A) B220^+^IgM^a+^cells were gated as indicated in S1 Fig (see also panel B of this Fig) and the proportion of T1 (CD24^hi^CD21^-^), T2 (CD24^hi^, CD21^int^), follicular (CD24^lo^, CD21^+^; Fo) and marginal zone/precursor (CD24^int^, CD21^hi^; MZ/P) B cells determined based upon staining with anti-CD21 and anti-CD24, as shown in the cartoon on the right side of the Fig. Representative flow plots for the indicated mouse strains are shown on the left with the proportion of cells within each region given adjacent to each region. (B) Representative flow plots showing B220 and IgM^a^ expression following incubation of B6 or c1(96–100), IgTg or DTg, splenocytes with media alone or containing anti-IgM F(ab’)_2_ (10 μg/ml) or HEL (100 ng/ml) for 18 hours. Regions used to gate B220^+^IgMa^+^ cells or all B220^+^ cells (for IgTg cells incubated with anti-IgM) for Fig 2D are indicated.
(TIF)

S3 Fig Mixed chimeric mice show similar numbers of B6.CD45.1 and c1(96–100).CD45.2 plasma cells in the spleen. Immunofluorescent imaging of IgM^a+^ plasma cells within the red pulp and marginal zones of an F1(B6.CD45.1 x c1(96–100)) sHEL mouse. Spleen sections (5 μm) were stained with biotinylated-B220 (blue), anti-CD45.1 or -CD45.2 (green), and anti-IgM^a^ (red), with streptavidin-AMCA as the secondary stain. Magnification of inset images is 10x, while magnification of larger images and individual stains is 20x. Arrows indicate the location of specific plasma cells.
(TIF)

S4 Fig Immature B cell functional changes seen in c1(70–100) IgTg mice are similar to those seen in c1(96–100) IgTg mice. Bone marrow cells from B6.IgTg or c1(70–100).IgTg mice were cultured in the presence of IL-7 for 5 days to produce naïve immature B cells. (A) Calcium mobilization in immature IgTg B cells from B6 (red) and c1(70–100) (blue) mice was measured by flow cytometry. Cultured immature B cells were labeled with Indo-1 and cross-linked with anti-IgM Ab. (B&C) Cultured immature B cells from B6.IgTg (open circles) or c1(70–100).IgTg (filled circles) mice were stimulated in media alone or containing anti-IgM F(ab’)_2_ or HEL (at various concentrations) for 20 hours. Cells were then stained with B220 and PI, and analyzed by flow cytometry. Scatterplots show the percentage of specific B cell death ((% PI^+^ with anti-IgM or HEL—% PI^+^ with media alone) divided by the % PI^-^ cells with media alone x100). Each circle represents the result from an individual mouse with the mean indicated by the lines. The asterisks indicate p values <0.05 (*) or <0.001 (**). Statistical analyses were performed using the Mann-Whitney *U* test.
(TIF)

S1 Table Comparison of splenic pre-immune B cell subsets in B6, c1(96–100), and c1(70–100) DTg mice. (DOCX)
