# Supplementary figures and images for: Multiple tolerance defects contribute to the breach of B cell tolerance in New Zealand Black chromosome 1 congenic mice
Source: PLoS One. 2017 Jun 19;12(6):e0179506. doi: 10.1371/journal.pone.0179506 (PMC5476272; doi:10.1371/journal.pone.0179506)

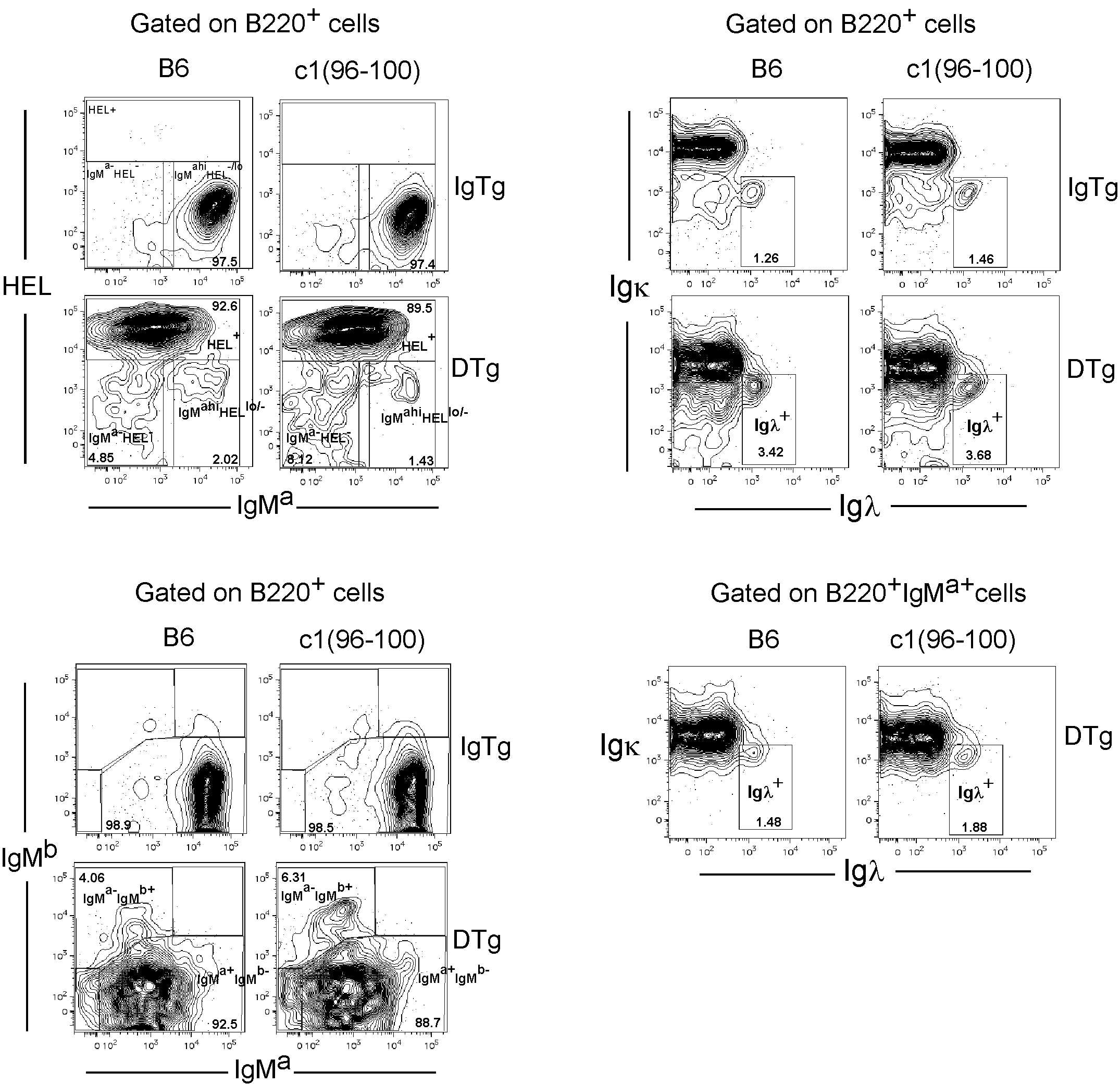

Supplement: S1 Fig — Splenocytes were stained as outlined in the materials and methods, and dead cells excluded by PI staining. Labels at the top of each cluster of graphs indicated the gated cells. Boxes in each plot show the regions used to identify various populations of B cells (corresponding to those in Table 1) with the specific populations being indicated on the plots for the DTg splenocytes. Numbers in the regions indicate the percentage of each population within the gated cells. (TIF) [file pone.0179506.s001.tif]

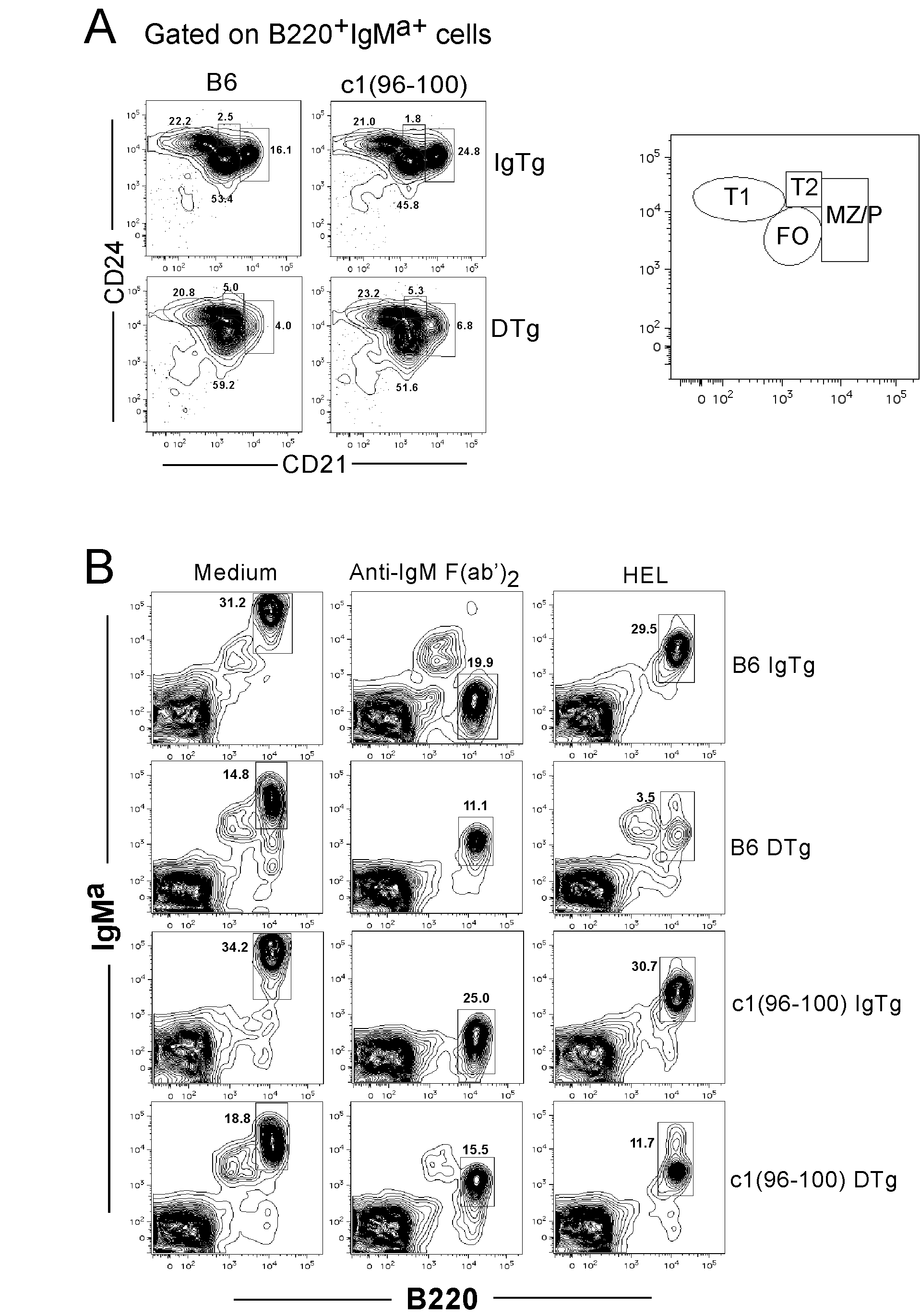

Supplement: S2 Fig — (A) B220+IgMa+cells were gated as indicated in S1 Fig (see also panel B of this Fig) and the proportion of T1 (CD24hiCD21-), T2 (CD24hi, CD21int), follicular (CD24lo, CD21+; Fo) and marginal zone/precursor (CD24int, CD21hi; MZ/P) B cells determined based upon staining with anti-CD21 and anti-CD24, as shown in the cartoon on the right side of the Fig. Representative flow plots for the indicated mouse strains are shown on the left with the proportion of cells within each region given adjacent to each region. (B) Representative flow plots showing B220 and IgMa expression following incubation of B6 or c1(96–100), IgTg or DTg, splenocytes with media alone or containing anti-IgM F(ab’)2 (10 μg/ml) or HEL (100 ng/ml) for 18 hours. Regions used to gate B220+IgMa+ cells or all B220+ cells (for IgTg cells incubated with anti-IgM) for Fig 2D are indicated. (TIF) [file pone.0179506.s002.tif]

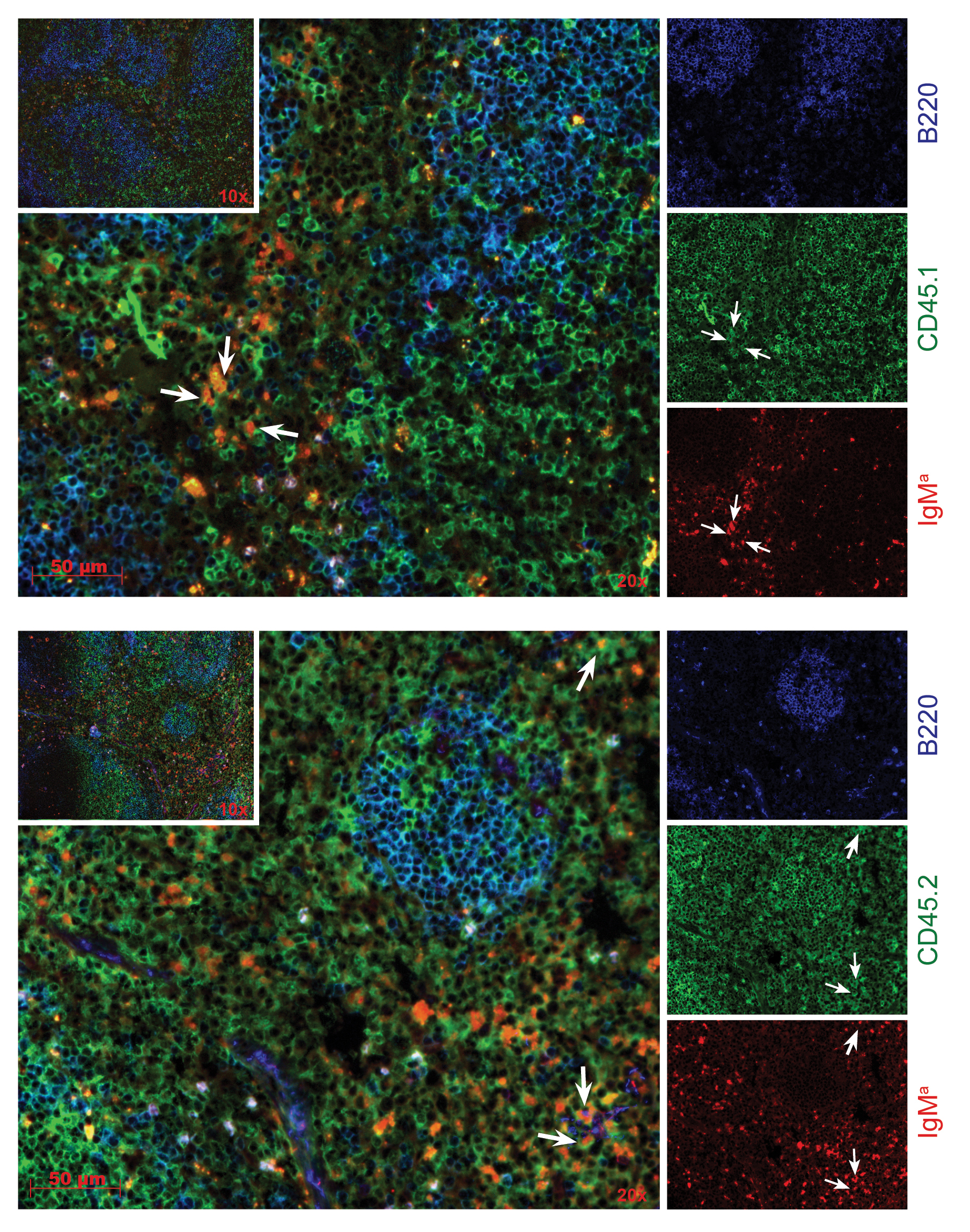

Supplement: S3 Fig — Immunofluorescent imaging of IgMa+ plasma cells within the red pulp and marginal zones of an F1(B6.CD45.1 x c1(96–100)) sHEL mouse. Spleen sections (5 μm) were stained with biotinylated-B220 (blue), anti-CD45.1 or -CD45.2 (green), and anti-IgMa (red), with streptavidin-AMCA as the secondary stain. Magnification of inset images is 10x, while magnification of larger images and individual stains is 20x. Arrows indicate the location of specific plasma cells. (TIF) [file pone.0179506.s003.tif]

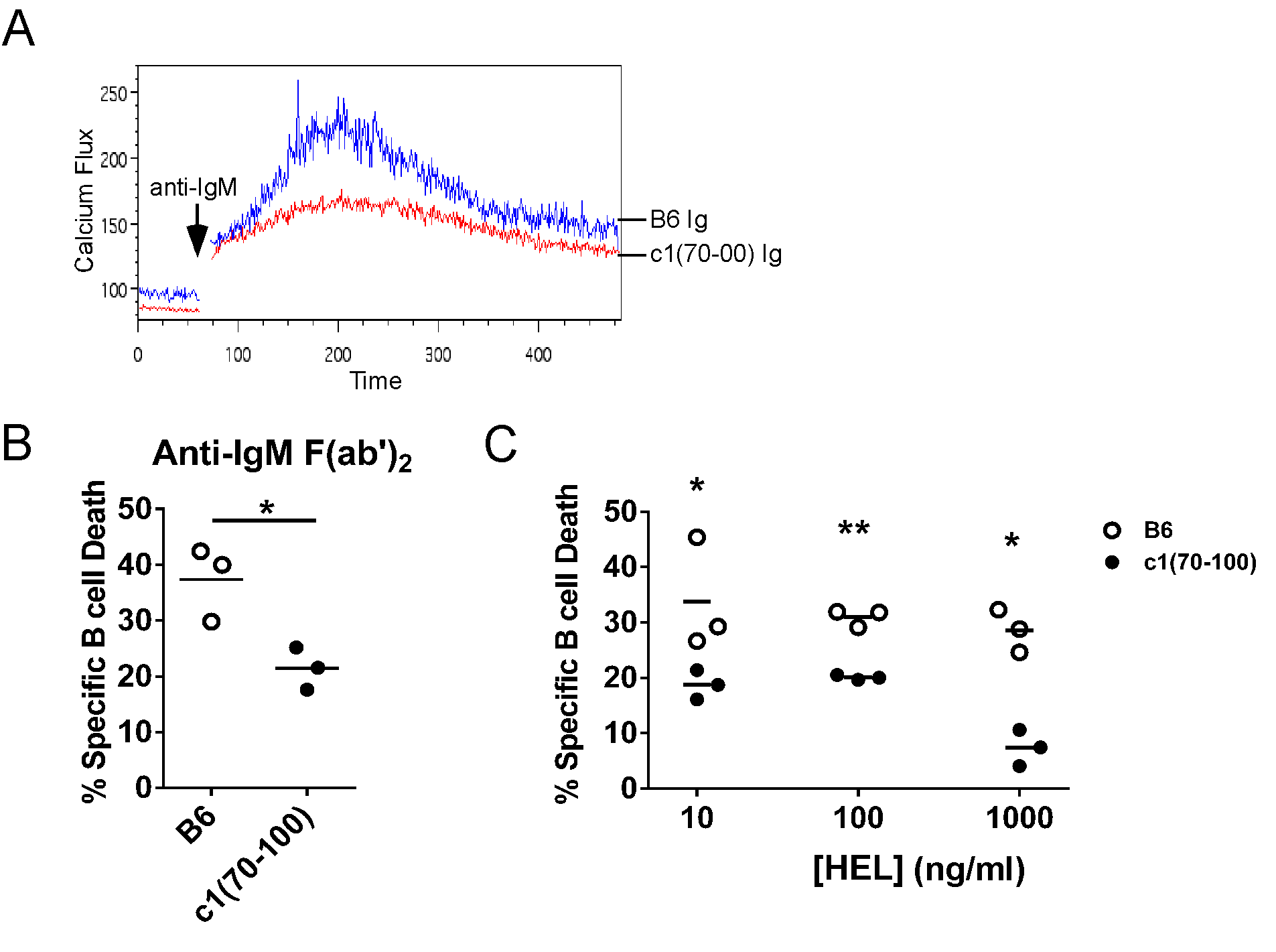

Supplement: S4 Fig — Bone marrow cells from B6.IgTg or c1(70–100).IgTg mice were cultured in the presence of IL-7 for 5 days to produce naïve immature B cells. (A) Calcium mobilization in immature IgTg B cells from B6 (red) and c1(70–100) (blue) mice was measured by flow cytometry. Cultured immature B cells were labeled with Indo-1 and cross-linked with anti-IgM Ab. (B&C) Cultured immature B cells from B6.IgTg (open circles) or c1(70–100).IgTg (filled circles) mice were stimulated in media alone or containing anti-IgM F(ab’)2 or HEL (at various concentrations) for 20 hours. Cells were then stained with B220 and PI, and analyzed by flow cytometry. Scatterplots show the percentage of specific B cell death ((% PI+ with anti-IgM or HEL—% PI+ with media alone) divided by the % PI- cells with media alone x100). Each circle represents the result from an individual mouse with the mean indicated by the lines. The asterisks indicate p values <0.05 (*) or <0.001 (**). Statistical analyses were performed using the Mann-Whitney U test. (TIF) [file pone.0179506.s004.tif]
