# Supplementary material for: Multiple tolerance defects contribute to the breach of B cell tolerance in New Zealand Black chromosome 1 congenic mice
Source: PLoS One. 2017 Jun 19;12(6):e0179506. doi: 10.1371/journal.pone.0179506 (PMC5476272; doi:10.1371/journal.pone.0179506)
Supplement: S1 Table — (DOCX) [file pone.0179506.s005.docx]

|  | **B6 DTg**  **n=10** | **c1(96-100) DTg**  **n=7** | **c1(70-100) DTg**  **n=19** |
| --- | --- | --- | --- |
| **% of B220^+^ cells** |  |  |  |
| **IgM^a+^HEL^+^** | 92.99±1.75 | 87.20±6.32 | 89.64±5.72 |
| **IgM^a-^HEL^-^** | 3.24±1.48 | 5.86±2.60 | 6.08±3.84 |
| **IgM^ahi^HEL^lo/-^** | 0.92±0.77 | 1.70±1.49 | 0.97±0.82 |
| **IgM^a+^IgM^b-^** | 84.52±5.83 | 85.27±3.87 | 81.89±8.15 |
| **IgM^a-^IgM^b+^** | 11.19±6.94 | 9.27±3.21 | 12.78±7.05 |
| **Igλ^+^** | 4.11±3.08 | 5.52±2.59 | 5.42±3.16 |
| **Igλ^+^IgM^a+^** | 4.27±4.31 | 5.37±4.48 | 5.28±3.61 |
|  |  |  |  |
| **% of IgM^a+^B220^+^cells** |  |  |  |
| **T1** | 34.71±9.99 | 29.40±17.65 | 33.63±6.15 |
| **T2** | 9.75±4.16 | 8.64±3.11 | 9.10±2.38 |
| **MZ/P** | 2.76±1.02 | 7.44±5.01***** | 7.86±6.01***** |
| **Fo** | 45.83±11.51 | 47.40±17.91 | 41.82±9.03 |
| **MFI (x10^3^) of IgM^a^** | 3.69±2.15 | 4.37±2.08 | 4.11±2.70 |

**Table S1.** Comparison of splenic pre-immune B cell subsets in B6, c1(96-100), and c1(70-100) DTg mice

Cell populations were gated as shown in Figures S1 and S2A. Results shown are mean ± standard deviation. Significance levels were determined by the Mann-Whitney U test, with significant differences (p<0.05) from B6 shown by asterisks. There were no significant differences between c1(96-100) and c1(70-100) DTg mice. The cohorts of B6 and c1(96-100) mice examined we independent from those in Figures 1 and 2.
